# At what times during infection is SARS-CoV-2 detectable and no longer detectable using RT-PCR-based tests? A systematic review of individual participant data

**DOI:** 10.1186/s12916-020-01810-8

**Published:** 2020-11-04

**Authors:** Sue Mallett, A. Joy Allen, Sara Graziadio, Stuart A. Taylor, Naomi S. Sakai, Kile Green, Jana Suklan, Chris Hyde, Bethany Shinkins, Zhivko Zhelev, Jaime Peters, Philip J. Turner, Nia W. Roberts, Lavinia Ferrante di Ruffano, Robert Wolff, Penny Whiting, Amanda Winter, Gauraang Bhatnagar, Brian D. Nicholson, Steve Halligan

**Affiliations:** 1 Centre for Medical Imaging, University College London, 2nd Floor, Charles Bell House, 43-45 Foley Street, London, W1W 7TS UK; 2 NIHR In Vitro Diagnostics Co-operative, Newcastle University, Newcastle upon Tyne, NE2 7RU UK; 3 NIHR In Vitro Diagnostics Co-operative, Newcastle upon Tyne Hospitals NHS Foundation Trust, Newcastle upon Tyne, NE7 7DN UK; 4 Exeter Test Group, Institute of Health Research, University of Exeter Medical School, University of Exeter, College House, St Luke’s Campus, Exeter, EX1 2LU UK; 5 Test Evaluation Group, Academic Unit of Health Economics, Leeds Institute of Health Sciences, University of Leeds, Worsley Building , Clarendon Way, Leeds, LS2 9LJ UK; 6 Nuffield Department of Primary Care Health Sciences, University of Oxford, Oxford, UK; 7 Cancer Services, Gastroenterology, Population Health & Primary Care, Bodleian Health Care Libraries, University of Oxford, Oxford, OX2 6HT UK; 8 Test Evaluation Research Group, Institute of Applied Health Research, University of Birmingham, Birmingham, B15 2TT UK; 9 Kleijnen Systematic Reviews Ltd, York, UK; 10 Bristol Medical School, University of Bristol, Bristol, UK; 11 Frimley Health NHS Foundation Trust, Frimley, Camberley GU16 7UJ UK

**Keywords:** SARS-CoV-2, RT-PCR, COVID-19, QUADAS-2, Diagnostic test, Anatomical sampling, IPD, Duration virus detection, Systematic review

## Abstract

**Background:**

Tests for severe acute respiratory syndrome coronavirus 2 (SARS-CoV-2) viral ribonucleic acid (RNA) using reverse transcription polymerase chain reaction (RT-PCR) are pivotal to detecting current coronavirus disease (COVID-19) and duration of detectable virus indicating potential for infectivity.

**Methods:**

We conducted an individual participant data (IPD) systematic review of longitudinal studies of RT-PCR test results in symptomatic SARS-CoV-2. We searched PubMed, LitCOVID, medRxiv, and COVID-19 Living Evidence databases. We assessed risk of bias using a QUADAS-2 adaptation. Outcomes were the percentage of positive test results by time and the duration of detectable virus, by anatomical sampling sites.

**Results:**

Of 5078 studies screened, we included 32 studies with 1023 SARS-CoV-2 infected participants and 1619 test results, from − 6 to 66 days post-symptom onset and hospitalisation. The highest percentage virus detection was from nasopharyngeal sampling between 0 and 4 days post-symptom onset at 89% (95% confidence interval (CI) 83 to 93) dropping to 54% (95% CI 47 to 61) after 10 to 14 days. On average, duration of detectable virus was longer with lower respiratory tract (LRT) sampling than upper respiratory tract (URT). Duration of faecal and respiratory tract virus detection varied greatly within individual participants. In some participants, virus was still detectable at 46 days post-symptom onset.

**Conclusions:**

RT-PCR misses detection of people with SARS-CoV-2 infection; early sampling minimises false negative diagnoses. Beyond 10 days post-symptom onset, lower RT or faecal testing may be preferred sampling sites. The included studies are open to substantial risk of bias, so the positivity rates are probably overestimated.

## Background

Accurate testing is pivotal to controlling severe acute respiratory syndrome coronavirus 2 (SARS-CoV-2), otherwise known as the coronavirus disease 2019 (COVID-19).

Considerable political and medical emphasis has been placed on rapid access to testing both to identify infected individuals so as to direct appropriate therapy, appropriate return to work, and to implement containment measures to limit the spread of disease. However, success depends heavily on test accuracy. Understanding when in the disease course the virus is detectable is important for two purposes, firstly to understand when and how to detect SARS-CoV-2, and secondly to understand how long individuals are likely to remain infective posing a risk to others.

The success of COVID-19 testing depends heavily on the use of accurate tests at the appropriate time. Testing for active virus infection relies predominantly on reverse transcription polymerase chain reaction (RT-PCR), which detects viral ribonucleic acid (RNA) that is shed in varying amounts from different anatomical sites and at different times during the disease course. It is increasingly understood that differences in virus load impact directly on diagnostic accuracy, notably giving rise to negative tests in disease-positive individuals [1, 2].

Positivity is contingent upon sufficient virus being present to trigger a positive test which may depend on test site, sampling methods, and timing [3]. For example, it is believed that positive nasopharyngeal RT-PCR declines within a week of symptoms so that a positive test later in the disease course is more likely from sputum, bronchoalveolar lavage fluid, or stool [4]. Nomenclature for anatomical site is also unclear, with a wide variety of overlapping terms used such as “oral”, “throat”, “nasal”, “pharyngeal”, and “nasopharyngeal”.

Because testing is pivotal to management and containment of COVID-19, we performed an individual participant data (IPD) systematic review of emerging evidence about test accuracy by anatomical sampling site to inform optimal sampling strategies for SARS-CoV-2. We aimed to examine at what time points during SARS-CoV-2 infection it is detectable at different anatomical sites using RT-PCR-based tests.

## Methods

This IPD systematic review followed the recommendations of the PRISMA-IPD checklist [5].

### Eligibility

Eligible articles were any case series or longitudinal studies reporting participants with confirmed COVID-19 tested at multiple times during their infection and provided IPD for RT-PCR test results at these times. We stipulated that test timings were linked to index dates of time since symptom onset or time since hospital admission as well as COVID-19 diagnosis by positive RT-PCR and/or suggestive clinical criteria, for example World Health Organization (WHO) guidelines [6].

### Search strategy and article selection

Search strings were designed and conducted subsequently in PubMed, LitCOVID, and medRxiv by an experienced information specialist (NR). The search end date was 24 April 2020. We additionally included references identified by COVID-19: National Institute for Health Research (NIHR) living map of living evidence (http://eppi.ioe.ac.uk/COVID19_MAP/covid_map_v4.html), COVID-19 Living Evidence (https://ispmbern.github.io/covid-19/living-review/) with a volunteer citizen science team, “The Virus Bashers” (Additional file 1: Table S1).

### Data extraction

Data were extracted into pre-specified forms. We did not contact authors for additional information. Study, participant characteristics, and ROB were extracted in Microsoft Excel (KG, JS, SG, JA, AW, SM). Data included country, setting, date, number of participants and IPD participants, inclusion criteria, IPD selection, participant age, sample types, RT-PCR test type and equipment, and primers. RT-PCR test results were extracted using Microsoft Access (SM, BS, JP, ZZ, CH).

### Risk of bias

We could not identify an ideal risk of bias (ROB) tool for longitudinal studies of diagnostic tests, so we adapted the risk of bias tool for diagnostic accuracy studies QUADAS-2 [7] to include additional signalling questions to cover anticipated issues. ROB signalling questions, evaluation criteria, and domain assessment of potential bias are reported (Additional file 1: Table S2).

### Sampling method and grouping

Details of sampling sites and methods, including location of the sampling site(s) and any sample grouping (for example, if combined throat and nasal swabs), were extracted from full texts by a clinician (NS) with queries referred to a second clinician (ST). If stated, details of sampling methodology were recorded, including who collected samples, information regarding anatomical location (e.g. how the nasopharynx was identified), and sample storage (Additional file 1: Table S3).

### RT-PCR test result conversion to binary results

IPD RT-PCR results were extracted from each article and converted to binary results (“positive” or “negative”). Data from Kaplan-Meier (KM) curves were extracted using Web digitizer [8] (Additional file 1: Table S3).

### Data analysis

Days since symptom onset and days since hospital admission were calculated from reported IPD. Data were presented collated across 5-day time intervals for each sample method, with longer times grouped within the longest time interval, and 95% CI was calculated for proportions. For comparison of duration of positive RT-PCR from respiratory tract (RT) and faecal samples, analysis and graphical presentation were restricted to participants sampled by both methods. Data analysis used STATA (14.2 StataCorp LP, Texas, USA) (Additional file 1: Table S3).

## Results

### Included studies

**Fig. 1:**
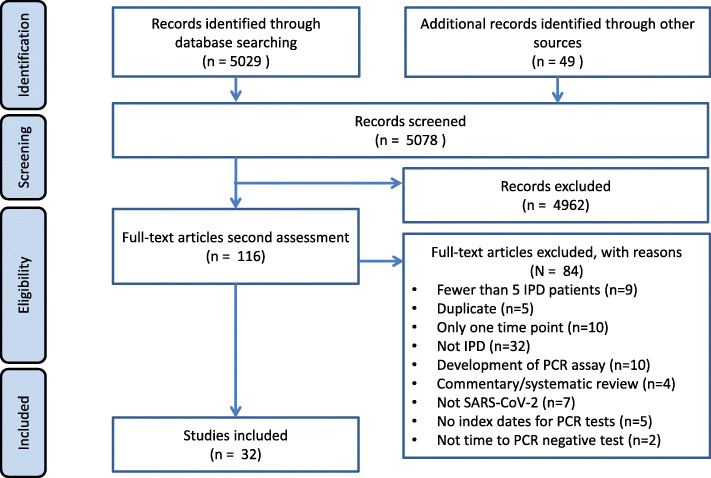
PRISMA flowchart

A total of 5078 articles were identified, 116 full text articles were screened, and 32 articles were included [9–40] (Fig. 1). Most articles were from China, in hospitalised adult participants (Table 1). Articles reported on a total of 1023 participants and 1619 test results.

**Table 1:** Study characteristics of study design

Ref	Author year	Country	Location	Setting	Date recruited	No. of participants in study	No. of IPD participants	Study design	Inclusion criteria: reference test
[9]	Cai 2020a	China	Wenzhou	Community	18 December 2019–12 February 2020	35	34	Contact tracing, all patients visited a shopping mall (confirmed COVID-19 cases that had contact with each other)	Positive RT-PCR test
[16]	Chang 2020	China	PLA General Hospital, Beijing	Hospital	28 January 2020 to 9 February 2020	16	16	Confirmed COVID-19 patients released from the Treatment Center of PLA General Hospital	Positive RT-PCR test from throat swabs
[10]	Chen 2020a	China	Beijing Ditan Hospital, Capital Medical University	Hospital	20 January to 27 February	133	22	Retrospectively identified a convenience sample of patients admitted to hospital	At least 2 positive RT-PCR test pharyngeal swabs
[12]	Chen 2020b	China	Shanghai, Shanghai Public Health Clinical Center.	Public Health Clinical Center	20 January 2020 to 6 February 2020	249	248	Retrospective cohort of laboratory confirmed cases	Positive RT-PCR test according to WHO interim guidelines
[29]	He 2020	China	Guangzhou Eighth People’s Hospital, Guangzhou, China	Hospital	21 January 2020 to 14 February 2020	94	19	All admitted COVID confirmed cases admitted to hospital	At least one positive RT-PCR test throat sample
[24]	Hu 2020a	China	Qingdao, Shandong	Hospital	29 January 2020 to 12 March 2020	59	59	Retrospective cohort of confirmed hospital cases	Positive RT-PCR test
[30]	Hu 2020b	China	Second Hospital of Nanjing, China	Hospital	28 January 2020 to 9 February 2020	24	23	Initial asymptomatic patients	Positive RT-PCR test and/or positive CT
[28]	Cai 2020b	China	Shanghai, China	Hospital	19 January 2020 to 3 February 2020	10	10	10 children admitted to hospital	Positive RT-PCR test nasopharyngeal or throat swab
[31]	Kujawski 2020	USA	Multiple sites, USA	Community	20 January 2020 to 5 February 2020	12	12	First 12 patients confirmed SARS-CoV-2 positive in the USA	Positive RT-PCR test in ≥ 1 specimen from a patient. Confirmed by CDC
[39]	Lavezzo 2020	Italy	Padua, Veneto region, Italy	Community	21 February 2020 to 7 March 2020	2812	80	Epidemiological study of entire population of Vo’, Italy, during complete closure of population movement	Positive RT-PCR test on nasopharyngeal swab
[27]	Lescure 2020	France	Paris, Bordeaux	Hospital	24 January 2020 to 29 January 2020	5	5	First 5 patients in France, exposed to COVID-19 in Hubei Province, China, prior to travel to France	Positive RT-PCR test
[34]	Li 2020	China	Xuzhou	Community	13 January 2020 to 17 February 2020	7	7	Asymptomatic contact tracing	Positive RT-PCR test
[14]	Liu 2020a	China	Hospital of Nanchang University	Hospital	21 January 2020 to 4 February 2020	76	25	Patients admitted to the hospital	Positive RT-PCR test from nasal swabs
[32]	Liu 2020b	China	Xixi Hospital of Hangzhou	Hospital	22 January 2020 to 11 February 2020	10	10	Hospital admission	Two consecutive positive RT-PCR tests
[18]	Lo 2020	China	Centro Hospitalar Conde de Sao Januario, Macau SAR, China	Hospital	21 January 2020 to 16 February 2020	10	10	Retrospective study of first 10 COVID-confirmed patients in Macau	Positive RT-PCR test
[20]	Lu 2020	China	Nantong, Nantong Third Hospital	Hospital	23 January 2020 to 6 March 2020	36	35	Retrospective analysis of clinical patients with fever, coughing, or lung inflammation	Positive RT-PCR test
[19]	Song 2020	China	Wuhan No. 1 Hospital, Nanjing Medical University	Hospital	31 January to 14 March 2020	13	13	Confirmed by CT images	Positive RT-PCR test
[13]	To 2020	China (Hong Kong)	Public Health Lab services branch in Hong Kong	Public Health Lab	Not reported	12	12	12 patients who presented with fever and acute respiratory distress, or pneumonia, and travel to Wuhan 14 days before onset of symptoms	Positive RT-PCR test from nasopharyngeal or sputum sample
[17]	Wolfel 2020	Germany	Munich, Germany	Hospital	Not reported	9	9	Patients that acquired infections upon known close contact to an index case (part of a larger cluster of epidemiologically linked cases that occurred after 23 January 2020 in Munich, Germany)	Positive RT-PCR test from oro- or nasopharyngeal swab
[37]	Wu 2020	China	Zhuhai, Guangdong	Hospital	16 January 2020 to 15 March 2020	74	41	Retrospective study of hospitalised patients	Positive RT-PCR test in two sequential respiratory tract samples
[40]	Wyllie 2020	USA	New Haven, CT	Community (healthcare workers)	Not reported to 5 April 2020	98 (44 inpatients)	19	Study of inpatients (44) and healthcare workers (98)	Positive RT-PCR test by nasopharyngeal swab or saliva
[21]	Xia 2020	China	Zhejiang	Hospital	26 January 2020 to 9 February 2020	30	30	A prospective interventional case series study of confirmed novel coronavirus pneumonia (NCP) patients at hospital	Positive RT-PCR test
[23]	Xiao 2020	China	Tongji Hospital, Huazhong, Wuhan	Hospital	21 January 2020 to 12 February 2020	56	56	30 confirmed novel coronavirus pneumonia (NCP) patients were selected	Positive RT-PCR test
[33]	Xu 2020a	China	Changzhou	Hospital	23 January 2020 to 27 February 2020	51	48	Hospital admission including 12 family clusters	Confirmed COVID-19 by Chinese diagnosis and treatment guideline
[36]	Xu 2020b	China	Guangzhou, Guangzhou Women and Children’s Medical Center	Hospital but from community surveillance	22 January 2020 to 20 February 2020	10	10	“Highly suspected” children with contact with confirmed COVID-19 person, or member of infected family group who was placed in quarantine	Positive RT-PCR test from nasopharyngeal or rectal swabs
[11]	Yang 2020	China	Shenzhen Third People’s	Hospital	11 January and 3 February 2020	213	13	COVID-19 positive hospitalised patients	Positive RT-PCR test
[26]	Young 2020	Singapore	4 hospitals in Singapore	Hospital	23 January 2020 to 3 February 2020	18	18	Contact tracing. First hospitalised patients in Singapore	Positive RT-PCR test
[22]	Yuan 2020	China	Fifth Affiliated Hospital, Zhuhai, Guangdong Province, China	Hospital	Late January	6	6	Case series	Positive RT-PCR test
[25]	Zhang 2020a	China	Wuhan Pulmonary Hospital, CAS Key Laboratory of Special Pathogens	Hospital	Not reported	Unclear	16	Prospective cohort patients	Positive RT-PCR test from oral swab
[35]	Zhang 2020b	China	Guangdong	Hospital	30 January 2020 to 5 February 2020	7	7	Prior hospitalisation for COVID-19, discharged under quarantine and subsequently positive by RT-PCR again	Positive RT-PCR test
[38]	Zheng 2020	China	Zheijang, China	Hospital	19 January 2020 to 15 February 2020	96	96	Retrospective cohort of 96 consecutively confirmed hospital cases	Positive RT-PCR test
[15]	Zou 2020	China	Zhuhai, Guangdong	Community and hospital	7 January to 26 January 2020	18	17	2 family clusters of patients infected by SARS-CoV	Positive RT-PCR test. Assay from Chinese CDC

Twenty-six (81%) articles reported data on test results since the start of symptoms, and 23 (72%) since hospital admission. Sixteen studies including 22% (229/1023) of the participants reported both these time points: The median time between symptom onset and hospitalisation was 5 days (interquartile range (IQR) 2 to 7 days). The median number of participants per study was 22 (IQR 9 to 56, range 5 to 232), and the median number of RT-PCR test results per participant was 4 (IQR 2 to 9) (Table 2).

**Table 2:** Study characteristics of IPD participant, sample site, and test type

Ref	Author year	How were IPD participants selected?	Percentage of male	Age median (years)	Age mean (years)	Age range (years)	Sample site	PCR test type; name of equipment if reported	Genomic targets; primers reported/referenced/ included
[9]	Cai 2020	IPD of almost all patients in study	NR	NR	NR	NR	Not reported	qRT-PCR; not reported	ORF1ab, N-gene; not reported
[16]	Chang 2020	IPD of all patients in study	69	36	Not reported	IQR 24–43	Throat	qRT-PCR; not reported	Not reported; not reported
[10]	Chen 2020a	Initial or follow-up positive sputum or faecal samples paired with a follow-up negative pharyngeal sample	64	37	37	IQR 30–49	Faeces, sputum, URT (pharyngeal)	qRT-PCR; not reported	ORF1ab, N-gene; not reported
[12]	Chen 2020b	IPD of all patients in study	51	51		IQR 36–64	URT	qRT-PCR; not reported	ORF1ab, N-gene; not reported
[29]	He 2020	IPD for all 94 patients presented, but could only extract 19 IPD from overlapping graphs	50	47			Throat	qRT-PCR; not reported	N-gene; not reported
[24]	Hu 2020a	IPD of all patients in study	48	46	NR	NR	Nasopharyngeal	qRT-PCR; not reported	ORF1ab, N-gene; not reported
[30]	Hu 2020b	IPD of almost all patients in study	33	33	38	5–95	Throat	qRT-PCR; BGI Genomics	ORF1ab; primers included
[28]	Jiehao 2020	IPD of all patients in study	40	7	6	3 months–11 years	URT	qRT-PCR; not reported	ORF1ab, N-gene; not reported
[31]	Kujawski 2020	IPD of all patients in study	67	53		21–68	Throat, nasopharyngeal, sputum, urine, faeces	qRT-PCR; not reported	N-gene; not reported
[39]	Lavezzo 2020	All residents identified with infection	50	NR	NR	NR	Nasopharyngeal	qRT-PCR; One Step Real Time kit (Thermo Fisher Scientific, USA)	E-gene, RdRp; primers referenced
[27]	Lescure 2020	IPD of all patients in study	60	46	47	30–80	Nasopharyngeal, faeces, conjunctiva, urine, blood, LRT (pleural)	qRT-PCR; not reported	E-gene, RdRp; primers included
[34]	Li 2020	IPD of all patients in study	57	42	43	21–62	Throat	qRT-PCR; not reported	Not reported; not reported
[14]	Liu 2020a	25 participants with serial samples tested for PCR	NR	NA	NA	NA	Nasopharyngeal	qRT-PCR; not reported	Not reported; not reported
[32]	Liu 2020b	IPD of all patients in study	40	42		34–50	Mixed URT (nasal, throat)	qRT-PCR; not reported	E-gene, RdRp, N-gene; not reported
[18]	Lo 2020	IPD of all patients in study	30	54	NR	NR	Faeces, nasopharyngeal, urine	qRT-PCR; BioGerm	ORF1ab, N-gene; not reported
[20]	Lu 2020	IPD of all patients in study	NR	NR	NR	NR	Sputum, URT (pharyngeal), faeces, blood	qRT-PCR; not reported	ORF1ab, N-gene; primers included
[19]	Song 2020	IPD of all patients in study	100	NR	NR	22–67	URT (pharyngeal)	qRT-PCR; Huirui biotechnology	Not reported; not reported
[13]	To 2020	IPD of all patients in study	58	63		37 to 75	Saliva	qRT-PCR; QuantiNova SYBR Green RT-PCR kit (Qiagen)	S-gene; not reported
[17]	Wolfel 2020	IPD of all patients in study	NR	Not reported	Not reported	Not reported	Sputum, faeces, URT (pharyngeal)	qRT-PCR; Tib-Molbiol, Germany	E-gene, RdRp; not reported
[37]	Wu 2020	Retrospective 41 patients with positive faecal samples only	53	NR	NR	NR	Throat, faeces	qRT-PCR; 2019-nCOV Real Time RT-PCR kit (LifeRiver Ltd)	E-gene, RdRp, N-gene
[40]	Wyllie 2020	Patients with multiple nasopharyngeal swabs or multiple saliva swabs	Inpatients (52), healthcare workers (16)	NR	61	23–92	Nasopharyngeal, saliva	qRT-PCR; US CDC RT-PCR primer/probe sets	N-gene; primers referenced
[21]	Xia 2020	IPD of all patients in study	70	51	55	13–83	Sputum, conjunctiva	qRT-PCR; BioGerm	Not reported; not reported
[23]	Xiao 2020	IPD of all patients in study	61	55	55	25–83	URT (pharyngeal)	qRT-PCR; Shanghai Huirui Biotechnology Co.	ORF1ab, N-gene; not reported
[33]	Xu 2020a	IPD of almost all patients in study	49	3 groups: imported 35 [29–51], secondary 37 [24–47.5], tertiary 53 [35–65]		24–65	Throat	qRT-PCR; BioGerm	ORF1ab, N-gene; not reported
[36]	Xu 2020b	IPD of all patients in study	60	7	8	2 months–16 years	Nasopharyngeal, faeces	qRT-PCR; BioGerm	ORF1ab, N-gene; primers included
[11]	Yang 2020	Not reported how 13 patients for serial sampling IPD data chosen	51	52		2–86	Mixed URT (nasal swabs, throat swabs) and mixed LRT (sputum and bronchoalveolar lavage fluid (BALF))	qRT-PCR; GeneoDX Co.	Not reported; not reported
[26]	Young 2020	IPD of all patients in study	50	47		31–73	Nasopharyngeal, faeces, urine, blood	qRT-PCR; not reported	ORF1ab, N-gene, S-gene; primers included
[22]	Yuan 2020	IPD of all patients in study	33	64	59	36–71	Nasopharyngeal, faeces	qRT-PCR; not reported	E-gene, RdRp, N-gene; not reported
[25]	Zhang 2020a	Not reported	NR	NR	NR	NR	URT (oral), faeces	qRT-PCR; HiScript®II One Step qRT-PCR SYBR®Green Kit (Vazyme Biotech Co.)	S-gene; primer included
[35]	Zhang 2020b	IPD of all patients in study	86	26	22	10 months–35 years	Throat, faeces, blood	qRT-PCR; not reported	Not reported; not reported
[38]	Zheng 2020	IPD of all patients in study	60	55	NR	NR	Sputum, faeces, blood	qRT-PCR; SARS-CoV-2 detection kit (BoJie Shanghai)	ORF1ab; not reported
[15]	Zou 2020	IPD of almost all patients in study	50	59		26–76	Throat, URT (nasal)	qRT-PCR; not reported	ORF1ab, N-gene; not reported

### Sampling site reporting

Articles variably specified sampling sites according to anatomical location, or grouped more than one site for analysis, for example as upper RT (Additional file 1: Table S4). The most frequent sample sites were faeces (*n* = 13), nasopharyngeal (*n* = 10), and throat (*n* = 9), although there was a range of other sites including blood, urine, semen, and conjunctival swabs (Table 2). Details of sampling method were generally absent. Two studies specified the person taking the samples. One study described how the nasopharynx was identified and the swab technique (length of contact time with the nasopharynx and twisting). Five studies specified sample storage and transport details.

### Sampling site positivity over time

**Fig. 2:**
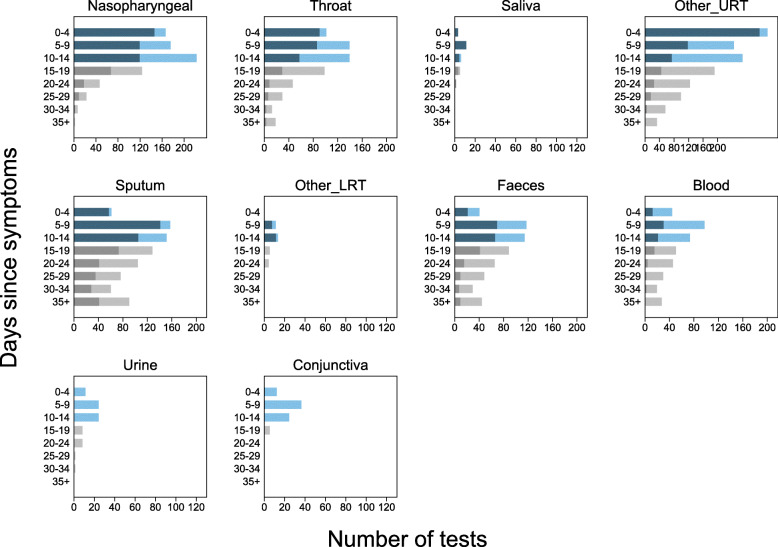
Number of positive and negative RT-PCR test results since symptom onset. Each panel shows a separate site used in participant sampling. Nasopharyngeal, saliva, and sputum were used where clearly reported. Throat included throat and oropharyngeal. Other URT includes samples reported in articles as nasal, mixed nasal and throat, oral, pharyngeal, or upper respiratory tract. For pharyngeal sampling, it was not clear if this was nasopharyngeal or oropharyngeal. Other LRT includes sampling reported as lower respiratory tract or one article including pleural fluid sampling. Blood included serum, plasma, or blood. Faeces included stool or anal swab. Each panel shows 5-day time periods since the onset of symptoms: 0–4 days, 5–9 days, 10–14 days, 15–19 days, 20–25 days, 26–30 days, 31–34 days, and 35 to max days. The numbers of positive RT-PCR tests are shown as dark blue bars and dark grey bars from 0 to 14 days and 15 to 40 days, respectively, and the number of negative RT-PCR results is shown similarly as light blue bars and light grey bars. Different colours are used before and after 15 days to indicate caution, as after 15 days testing is enriched in more severely ill participants. The total number of tests within a particular time period can be read from the *x*-axis

**Fig. 3:**
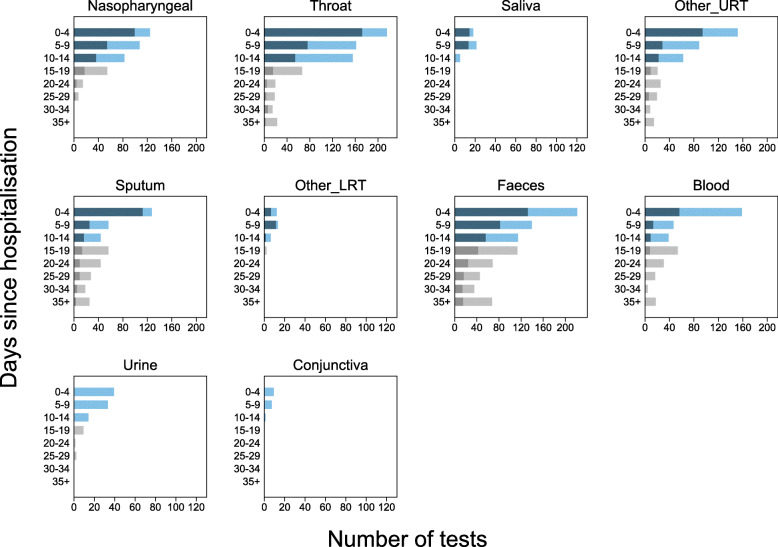
Number of positive and negative RT-PCR test results since hospital admission. Each panel shows a separate site used in participant sampling. Nasopharyngeal, saliva, and sputum were used where clearly reported. Throat included throat and oropharyngeal. Other URT includes samples reported in articles as nasal, mixed nasal and throat, oral, pharyngeal, or upper respiratory tract. For pharyngeal sampling, it was not clear if this was nasopharyngeal or oropharyngeal. Other LRT includes sampling reported as lower respiratory tract or one article including pleural fluid sampling. Blood included serum, plasma, or blood. Faeces included stool or anal swab. Each panel shows 5-day time periods since the hospital admission: 0–4 days, 5–9 days, 10–14 days, 15–19 days, 20–25 days, 26–30 days, 31–34 days, and 35 to max days. The numbers of positive RT-PCR tests are shown as dark blue bars and dark grey bars from 0 to 14 days and 15 to 40 days, respectively, and the number of negative RT-PCR results is shown similarly as light blue bars and light grey bars. Different colours are used before and after 15 days to indicate caution, as after 15 days testing is enriched in more severely ill participants. The total number of tests within a particular time period can be read from the *x*-axis

We present RT-PCR test results for 11 different sampling sites at different times during SARS-CoV-2 infection. Figures 2 and 3 show the number of positive and negative RT-PCR results for 5-day time intervals since symptom onset and time from hospital admission, respectively.

The sampling sites yielding the greatest proportion of positive tests were nasopharyngeal, throat, sputum, or faeces. Insufficient data were available to evaluate saliva and semen. Only 33% of participants who were tested with blood samples had detectable virus (44/133; 6 articles [20, 26, 27, 31, 35, 38]), and almost no samples tested from urine or conjunctival sampling detected virus presence.

Using nasopharyngeal sampling, 89% (147/166, 95% CI 83 to 93) RT-PCR test results were positive from 0 to 4 days post-symptom onset and 81% (100/124, 95% CI 73 to 87) 0 to 4 days post-hospital admission (Figs. 2 and 3). At 10 to 14 days, the percentage of test results positive reduced to 54% (120/222, 95% CI 47 to 61) post-symptoms and 45% (37/82, 95% CI 34 to 57) post-admission (Additional file 1: Figures S6 and S7).

Using throat sampling at 0 to 4 days post-symptoms, 90% (91/101, 95% CI 83 to 95) of test results from participants with SARS-CoV-2 were detected by RT-PCR sampling, falling to 42% (58/139, 95% CI 33 to 50) at 10 to 14 days post-symptom onset (Fig. 2, Additional File 1: Figures S6 and S7). Similar results were observed for time since hospital admission, where at 0 to 4 days 80% (173/215, 95% CI 75 to 86) of result were positive, falling to 35% (55/155, 95% CI 28 to 44) between 10 and 14 days (Fig. 3, Additional file 1: Figures S6 and S7). Using faecal sampling, 55% test results are positive (22/40, 95% CI 38 to 71) at 0 to 4 days post-symptom onset.

### Upper and lower respiratory tract sampling

**Fig. 4:**
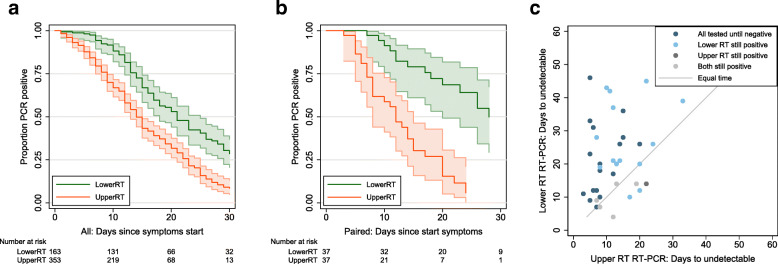
Comparison of duration of detectable virus from upper and lower respiratory tract sampling. **a** Time to undetectable virus in upper and lower respiratory tract samples. Kaplan-Meier with 95% confidence intervals and number at risk. All samples in review. **b** Time to undetectable virus in upper and lower respiratory tract samples in participants who were tested with both upper and lower respiratory tract sampling. Kaplan-Meier with 95% confidence intervals and number at risk. Restricted to participants with both sampling methods. **c** Time to undetectable virus in upper and lower respiratory tract samples in participants who were tested with both upper and lower respiratory tract sampling. Scatterplot where each dot represents a single participant, with the time to undetectable virus with both upper and lower respiratory tract sampling shown for each participant

We further grouped sites into upper (URT) and lower (LRT) respiratory tract. The rate of sample positivity reduced faster from URT sites compared to LRT sites (Fig. 4a). Given that analysis across all participants is likely to be influenced by preferential URT sampling of participants with less severe disease, we also analysed participants who underwent both URT and LRT sampling. Again, URT sites on average cleared faster (median 12 days, 95% CI 8 to 15 days) than LRT sites (median 28 days, 95% CI 20 to not estimable; Fig. 4b); the majority of participants clear virus from URT site before LRT (Fig. 4c). Data based on time since hospital admission are consistent with data for time since symptom onset.

### Faecal vs. respiratory tract sampling

**Fig. 5:**
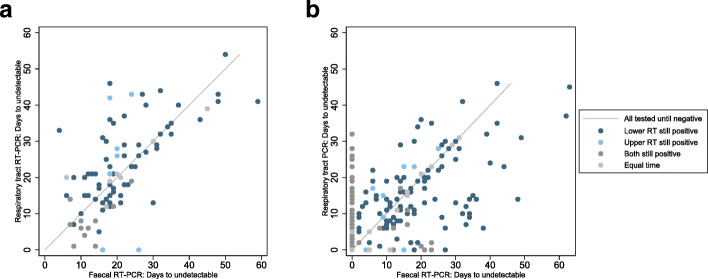
Comparison of days to undetectable virus from respiratory tract and faecal sampling. Time to undetectable virus in faecal compared to any respiratory tract sample in participants who were tested with both sampling. Scatterplot where each dot represents a single participant, with the time to undetectable virus with both faecal and respiratory tract sampling shown for each participant. Thirty percent of participants tested at both sampling sites do not have detectable virus in faecal samples

Across participants sampled by both RT and faecal sampling since hospital admission, 29% of participants were detected using RT sampling but not by faecal sampling (52/177 participants, 95% CI 23 to 37%, 10 studies). The time to RT-PCR tests becoming undetectable varied greatly by participant, although time to undetectable virus was similar for both sampling sites (Fig. 5), in participants with RT-PCR test results from both RT and faecal samples. Thirty-nine out of 89 participants (44%, 95% CI 33 to 55%) had a shorter duration of detection in faecal samples than in RT samples.

Median time to clearance from RT was shorter in participants based on time since hospitalisation (125 participants, *p* = 0.014), whilst similar in participants since onset of symptoms (87 participants, *p* = 0.15) (Additional file 1: Figures S8).

### Intermittent false negative results

**Fig. 6:**
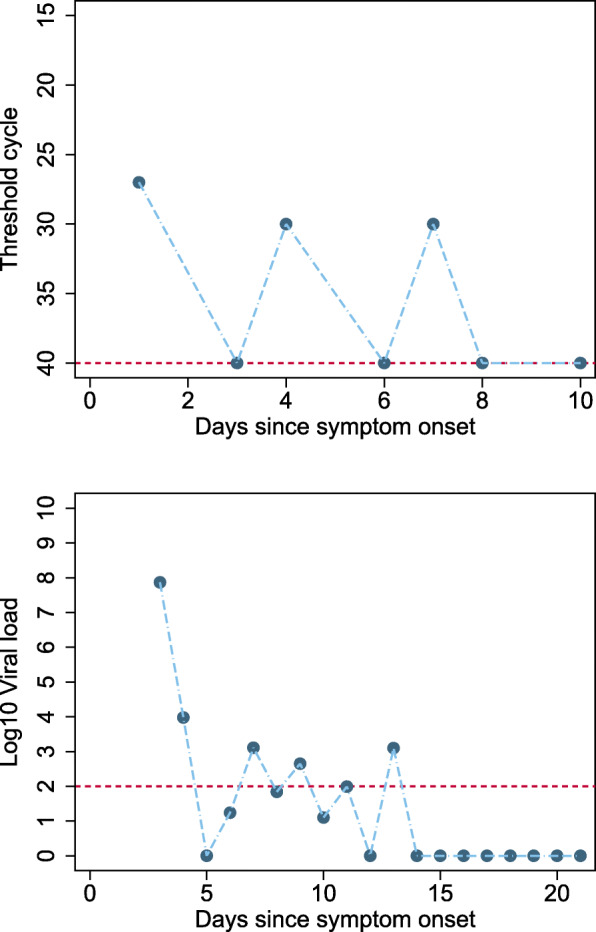
Example participants with intermittent false negative results. **a** An example of a participant with high viral load, but where alternate RT-PCR test results report high viral load or undetectable virus. **b **A participant where virus levels have reduced over time to a level around the limit of viral detection, and at these low levels of virus, intermittent negative results will occur due to differences in the location or amount of sample

Many articles reported intermittent false negative RT-PCR test results for participants within the monitoring time span. Where participant viral loads were reported, several different profiles were distinguished; two examples are shown in Fig. 6 [14, 15]. Intermittent false negative results were reported either where the level of virus is close to the limit of detection, or in participants with high viral load but for unclear reasons.

### Risk of bias

**Fig. 7:**
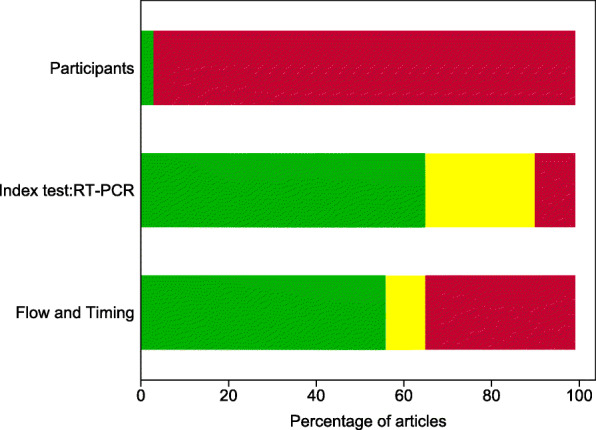
Risk of bias by adapted QUADAS-2 domain. An adapted version of QUADAS-2 for longitudinal studies was used (Additional file 1: Table S2). For each domain, the percentage of studies by concern for potential risk of bias is shown: low (green), unclear (yellow), and high (red)

The proportion of studies with high, low, or unclear ROB for each domain is shown in Fig. 7, and ROB for individual studies is shown in Additional file 1: Table S5. All studies were judged at high ROB. All but one were judged at high ROB for the participant selection domain [17], mainly as they only included participants with confirmed SARS-CoV-2 infection based on at least one positive PCR test. Studies also frequently selected a subset of the participant cohort for longitudinal RT-PCR testing, and only results for these participants were included in the study. Ten studies were judged at unclear ROB for the index test domain as the schedule of testing was based on clinician choice rather than being pre-specified by the study or clinical guidelines, or because the samples used for PCR testing were not pre-specified. Eleven studies were judged at high ROB for the flow and timing domain mainly because continued testing was influenced by easy access to participants, such as by continued hospitalisation.

## Discussion

### Key findings

Negative RT-PCR test results were common in people with SARS-CoV-2 infection confirming that RT-PCR testing misses identification of people with disease. Our IPD systematic review has established that sampling site and time of testing are key determinants of whether SARS-CoV-2 infected individuals are identified by RT-PCR.

We found that nasopharyngeal sampling was positive in approximately 89% (95% CI 83 to 93) of tests within 4 days of either symptom onset. Sampling 10 days after symptom onset greatly reduced the chance of a positive test result.

There were limited data on new methods of sample collection like saliva in these longitudinal studies. Sputum samples have similar or higher levels of detection to nasopharyngeal sampling, although this may be influenced by preferential sputum sampling in severely ill participants. Although based on few participants tested at both sampling sites, URT sites have faster viral clearance than LRT in most of these participants; 50% of participants were undetectable at URT sites 12 days after symptom onset compared to 28 days for LRT.

We found that faecal sampling is not suitable for initial detection of disease, as up to 30% of participants detected using respiratory sampling are not detected using faecal sampling. Viral detection in faecal samples may be useful to establish virus clearance, although as noted, whether RT or faecal samples have longer duration of viral detection varies between participants.

All included studies were judged at high ROB, so results of this review should be interpreted with caution. Table 3 provides an overview of the major methodological limitations and their potential impact on study results. A major source of bias is that all but one study [19] restricted inclusion to participants with confirmed SARS-CoV-2 infection based on at least one positive RT-PCR test, meaning that the percentage of positive RT-PCR testing is likely to be overestimated.

**Table 3:** Biases and issues in interpretation

Domain	Details of bias and applicability issues	Impact on interpretation of study data
Participants (source of bias)	In these studies, the reference test usually incorporates RT-PCR (index test). • RT-PCR testing is usually a key component of identifying people with SARS-CoV-2 infection. • Participants will not be detected or included in these studies when SARS-CoV-2 is not present at easily sampled sites and at the time that participants were available for testing.	Unclear how many and what severity of participants with SARS-CoV-2 are not included in studies. People who do not have a positive RT-PCR test at some point are excluded. This could lead to overestimation of positivity.
		Rates of positivity will be inflated as only people with virus accessible for sampling for RT-PCR tests will be included in studies.
Participants (source of bias)	Most participants are identified or present based on respiratory tract symptoms such as cough or respiratory distress.	Unclear how many and what severity of participants with SARS-CoV-2 are not included in studies.
	• Participants will not be detected or included in these studies when less common symptoms or asymptomatic.	
	• Participants included will be biased to over-represent people with detectable virus in respiratory tract sampling sites and at times frequently used for testing (post symptom onset or at admission to hospital).	Studies will inflate positivity for sampling sites that overlap with sampling sites used in RT-PCR reference testing. • For example, we identified 30% of participants with RT positivity but with negative results from faecal sampling. However, if participants had only faecal virus, would they have been included in the studies?
Index test: RT-PCR (applicability)	• Studies included are likely to use more invasive sampling methods than acceptable in widespread population testing. For example, nasopharyngeal testing is likely in many current studies to be based on long swabs and self test kits.	Percentage of people with detectable virus may be overestimated when testing is applied in real-world clinical use and in population testing.
	• Studies will use experienced staff to obtain samples, handle, process, and conduct tests.	
	• Studies are mostly sampling participants in hospital settings or in specialised research community testing research where sample handling, transport, and storage have been standardised.	
	• Variation in RT-PCR kits is minimised as studies are based in few hospitals or limited to a research setting	
Index test: RT-PCR (applicability)	Sample RNA extraction methods are designed predominantly for respiratory samples. • RT-PCR sample preparation kits used are mostly designed for extraction of virus from respiratory samples, not from faecal samples. It is unclear how efficiently these kits extract virus RNA from stool samples.	Percentage of people with virus present in faecal samples and duration of shedding in faecal samples may be underestimated.
Index test: RT-PCR (applicability)	RT-PCR tests detect both infectious and inactive (inactive due to immune system or dead) virus.	Percentage of people with clinically important detectable virus may be overestimated.
	• Few studies link RT-PCR testing to cell assays to test for infectious virus.	
Index test: RT-PCR (applicability)	Rate of virus aggregation or clearance by immune system may affect the sampling efficiency at some sampling sites. • Both the innate and adaptive immune system will aggregate and clear virus particles from bodily fluids. It is not clear what the time scale of clearance or how this influences detection of virus at different sites and at which time points.	Percentage of people with detectable virus may be underestimated.
Index test: RT-PCR (applicability)	Reporting of sampling sites and methods is poor. • Poor reporting may have led to less ideal grouping of sampling in analysis. • Some studies are likely to use a variety of nasopharyngeal sampling methods depending on the individual participants, but the type of sampling is typically reported at a study level for a particular sampling site.	Percentage of people with detectable virus may be over- or underestimated.
Flow and timing	Uncertainty and inconsistencies in time of sampling	Percentage of people with detectable virus may be over- or underestimated at particular times.
	• Time of symptom onset can be subjective unless based on fever, but some participants do not have fever. • Time of symptom onset may be different if asked of participants in ICU setting. • Time of hospitalisation and discharge may be affected by function hospitalisation serves in containment of disease spread. In some studies, the hospitals were also quarantine centres, so participants were hospitalised immediately at onset of mild symptoms rather than restricted to patients needing oxygen.	
Flow and timing	Clinical cohort within studies changes across time points.	Percentage of people with detectable virus may be overestimated at particular later time points as these correspond to participants who were severely ill.
	• Participants who have recovered from COVID-19 in most studies are typically not tested after 2 negative tests 24 h apart. • Many studies only test inpatients at the hospital, so the participants sampled between 0 and 14 days typically have less severe disease than those tested longer	
Flow and timing (selective outcome reporting)	Some studies only publish IPD data for a selection of people.	Available IPD data may not represent a typical spectrum of participants in the different settings (community setting, hospital, ICU, nursing home, prison).
Publication bias	Published data is likely to be biased towards publication of research active groups which may not represent typical real world.	Percentage of people with detectable virus may be overestimated.

*BAL* bronchoalveolar lavage, *COVID-19* coronavirus disease 2019, *ICU* intensive care unit, *IPD* individual participant data, *RNA* ribonucleic acid, *RT-PCR* reverse transcription polymerase chain reaction, *SARS-CoV-2* severe acute respiratory syndrome coronavirus 2

Lack of technical details, for example of how samples are taken and RT-PCR tests performed, limits the applicability of findings to current testing. Compared to real life, studies were likely to use more invasive sampling methods, use experienced staff to obtain samples, and sample participants in hospital settings where sample handling could be standardised. Consequently, estimates of test performance are likely to be overestimated compared to real-world clinical use and in community population testing including self-test kits.

These limitations have important implications for how testing strategies should be implemented and in particular how a negative RT-PCR test result should be interpreted.

### Putting the findings into context of literature

The accuracy of RT-PCR testing is limited by sampling sites used, methods, and the need to test as soon as possible from symptom onset in order to detect the virus. Previous studies have established that in COVID-19 infection, viral loads typically peak just before symptoms and at symptom onset [4] and estimated false negative test results over time since exposure from upper respiratory tract samples [2]. To our knowledge, there has been no prior systematic review of RT-PCR using IPD to quantify the percentage of persons tested who are positive and how this varies by time and sampling site.

Understanding the distribution of anatomical sites with detectable virus is clinically relevant, especially given independent viral replication sites in nose and throat using distinct and separate genetic colonies [17]. Understanding of different patterns of detection and duration of virus detection at different body sites is essential when designing strategies of testing to contain virus spread. Notably, it is unclear if detection of virus in faeces is important in disease transmission, although faecal infection was shown in SARS and MERS [41].

### Strengths of study

This review uses robust systematic review methods to synthesise published literature and identifies overall patterns not possible from individual articles. Using IPD, we examined data across studies and avoided study-level ecological biases present when using overall study estimates. IPD regarding sample site at different time points during infection is vital because it provides an overview of test performance impossible from individual studies alone. Synthesised IPD can also substantiate or reject patterns appearing within individual studies. Within-participant paired comparisons of sampling sites also become possible with sufficient data.

### Limitations of study

The main limitation is the risk of bias in the included studies. Although constraints were understandable given the circumstances in which the studies were done, the consequences for validity need to be highlighted. The percentage of positive RT-PCR testing is likely to be overestimated, because inclusion was restricted to participants with confirmed SARS-CoV-2 infection based on at least one positive RT-PCR test in all but one study [19]. This means that people who had a COVID-19 infection but never tested positive on at least one RT-PCR test would not have been included. This could arise if SARS-CoV-2 is not present at easily sampled sites or at the time participants were tested. This makes it impossible to determine the true false negative rate of the test—the proportion of people who actually have SARS-CoV-2 but would receive a negative RT-PCR test result. It is possible that only half of persons infected by SARS-CoV-2 may test positive, as a community surveillance study in Italy found only 53% (80/152) persons tested RT-PCR positive in households quarantined for 18 days with persons who tested PCR positive [39]. The same study also identified households where no one tested RT-PCR positive, but where there were clusters of persons with symptoms typical of COVID.

Poor reporting of sampling methods and sites impaired our ability to distinguish between and report on variability between them. For some sampling methods such as saliva and throat swabs, more studies are needed. There were also sparse data on sampling methods that are becoming more widespread, such as participant self-sampling [42] and short nasal swab sampling (anterior nares/mid turbinate) [43]. Our index times may be subject to bias as symptom onset is somewhat subjective and hospital admission practices vary by country, pandemic stage, and hospital role (i.e. healthcare vs. isolation). The results presented do not correspond to following the same participants across time, but the testing at clinically relevant time snapshots reported from individual studies, so that participants tested at later time points are likely to have more severe disease; this does not limit the interpretation of results in understanding testing of participants in most clinical contexts. Comparisons of sampling sites should be restricted to participants tested at the relevant sites.

We have used analysis methods that do not include clustering within studies, to keep analyses simple to understand and present, and to avoid complications of fitting models where the number of participants in each cluster varies. Ultimately, many potentially eligible studies did not report IPD which led to their exclusion, or only reported IPD for a subset of participants in the study. We would welcome contact and data sharing with clinicians and authors to rectify this.

### Implications for policy/practice/future research

To avoid the consequences of missed infection, samples for RT-PCR testing need to be taken as soon as symptoms start for detection of SARS-CoV-2 infection in preventing ongoing transmission.

Even within 4 days of symptom onset, some participants infected with SARS-CoV-2 will receive negative test results. Testing at later times will result in a higher percentage of false negative tests in people with SARS-CoV-2, particularly at upper RT sampling sites. After 10 days post-symptoms, it may be important to use lower RT or faecal sampling. Valid estimates are essential for clinicians interpreting RT-PCR results. However, ROB considerations suggest that the positive percentage rates we have estimated may be optimistic, possibly considerably so.

Participants can have detectable virus in different body compartments, so virus may not be detected if samples are only taken from a single site. Some hospitals in the UK now routinely take RT-PCR samples from multiple sites, such as the nose and throat. More studies are urgently needed on evolving sampling strategies such as self-collected samples which include saliva and short nasal swabs. Future studies should avoid the risks of bias we have identified by precisely reporting the anatomical sampling sites with a detailed methodology on sample collection. Table 4 details example studies helpful for future study design.

**Table 4:** Examples from included studies

Study characteristic	Detail	Reference
Study design: representative recruitment	Representative participants • Community infection • Contact tracing including asymptomatic • Hospitalised patients	Population surveillance of Italian town, with PCR testing across [39] Contact tracing [9, 30, 34] Retrospective cohort of 96 hospitalised patients [38]
Study design: planned testing and follow-up	Informative sampling • Multiple sampling sites per participant • Planned schedule of sampling • Sampling continues after negative test results • Sampling continues after hospital discharge	Three samples per patient, multiple testing including prolonged testing even after multiple negative results [10] Population surveillance of Italian town over 18 days [39] Long follow-up post-hospital [16, 35, 36] Planned schedule of testing ([30], asymptomatic contact tracing follow-up [36–38])
Study design: sampling	Reporting of sampling methods (sample site, staff conducting test, sample volumes, and methods)	Most studies had very sparse reporting of sample collection methods. Example of fuller reporting [40]
Individual participant data reporting for sharing	Examples of plots and tables that facilitated sharing of individual participant data	Retrospective cohort of 96 patients all tested with sputum, faeces, and blood. Plot shows time span of positive test results, hospitalisation timing, and disease severity for individual patients [38] Data showing time course of illness with PCR test results [9, 31] Data showing RT-PCR test results by patient and time point [10] Viral load over time [15]

Further sharing of IPD will be important, and we would welcome contact from groups with IPD data we can include in ongoing research.

## Conclusions

RT-PCR misses detection of people with SARS-CoV-2 infection; early sampling minimises false negative diagnoses. Beyond 10 days post-symptom onset, lower RT or faecal testing may be preferred sampling sites. The included studies are open to substantial risk of bias, so the positivity rates are probably overestimated.

## Supplementary information

**Additional file 1.** Including additional tables and figures: search details, QUADAS-2 adaption, anatomical sample size details, risk of bias by article, percentage positive and negative RT-PCR results by sample for days since symptom onset and days since hospitalisation, time to undetectable virus in faecal and respiratory tract.

## Data Availability

The datasets used and/or analysed during the current study are available from the corresponding author on reasonable request.

## Abbreviations

BAL: Bronchoalveolar lavage
BALF: Bronchoalveolar lavage fluid
CI: Confidence interval
Ct: Cycle threshold
HIV: Human immunodeficiency virus
IPD: Individual participant data
KM: Kaplan-Meier
LRT: Lower respiratory tract
MERS: Middle East respiratory syndrome
MeSH: Medical Subject Headings
NIHR: National Institute for Health Research
QUADAS-2: A Revised Tool for the Quality Assessment of Diagnostic Accuracy Studies
RNA: Ribonucleic acid
ROB: Risk of bias
RT-PCR: Reverse transcriptase polymerase chain reaction
RT: Respiratory tract
SARS: Severe acute respiratory syndrome
TOC: Table of characteristics
UK: United Kingdom
URT: Upper respiratory tract
WHO: World Health Organization

## References

[1] Wikramaratna P, Paton RS, Ghafari M, Lourenco J. Estimating false-negative detection rate of SARS-CoV-2 by RT-PCR. medRxiv. 2020; 2020.04.05.20053355. 10.2807/1560-7917.ES.2020.25.50.2000568 PMC7812420 33334398

[2] Kucirka LM, Lauer SA, Laeyendecker O, Boon D, Lessler J (2020). Variation in false-negative rate of reverse transcriptase polymerase chain reaction-based SARS-CoV-2 tests by time since exposure. Ann Intern Med.

[3] Yu F, Yan L, Wang N, Yang S, Wang L, Tang Y, et al. Quantitative detection and viral load analysis of SARS-CoV-2 in infected patients. Clin Infect Dis. 2020. 10.1093/cid/ciaa345 PMC7184442 32221523

[4] Sethuraman N, Jeremiah SS, Ryo A. Interpreting diagnostic tests for SARS-CoV-2. JAMA. 2020. 10.1001/jama.2020.8259 32374370

[5] Stewart LA, Clarke M, Rovers M, Riley RD, Simmonds M, Stewart G (2015). Preferred Reporting Items for Systematic Review and Meta-Analyses of individual participant data: the PRISMA-IPD Statement. JAMA..

[6] WHO. Clinical management of COVID-19. WHO Interim guidance. 2020;WHO/2019-nCoV/clinical/2020.5 (accessed 16 June2020).

[7] Whiting PF, Rutjes AW, Westwood ME, Mallett S, Deeks JJ, Reitsma JB (2011). QUADAS-2: a revised tool for the quality assessment of diagnostic accuracy studies. Ann Intern Med.

[8] Rohatgi A. WebPlotDigitizer https://apps.automeris.io/wpd/ (Accessed 16 June 2020). 2010.

[9] Cai J, Wenjie S, Jianping H, Michelle G, Jing W, Guiqing H (2020). Indirect virus transmission in cluster of COVID-19 cases, Wenzhou, China, 2020. Emerg Infect Dis.

[10] Chen C, Gao G, Xu Y, Pu L, Wang Q, Wang L, et al. SARS-CoV-2–positive sputum and feces after conversion of pharyngeal samples in patients with COVID-19. Ann Intern Med. 2020. 10.7326/M20-0991 PMC7133055 32227141

[11] Yang Y, Yang M, Shen C, Wang F, Yuan J, Li J, et al. Evaluating the accuracy of different respiratory specimens in the laboratory diagnosis and monitoring the viral shedding of 2019-nCoV infections. medRxiv. 2020; 2020.02.11.20021493.

[12] Chen J, Qi T, Liu L, Ling Y, Qian Z, Li T (2020). Clinical progression of patients with COVID-19 in Shanghai, China. J Infect.

[13] To KK, Tsang OT, Yip CC, Chan KH, Wu TC, Chan JM (2020). Consistent detection of 2019 novel coronavirus in saliva. Clin Infect Dis..

[14] Liu Y, Yan L-M, Wan L, Xiang T-X, Le A, Liu J-M (2020). Viral dynamics in mild and severe cases of COVID-19. Lancet Infect Dis.

[15] Zou L, Ruan F, Huang M, Liang L, Huang H, Hong Z (2020). SARS-CoV-2 viral load in upper respiratory specimens of infected patients. N Engl J Med.

[16] Chang D, Mo G, Yuan X, Tao Y, Peng X, Wang F-S (2020). Time kinetics of viral clearance and resolution of symptoms in novel coronavirus infection. Am J Respir Crit Care Med.

[17] Wölfel R, Corman VM, Guggemos W, Seilmaier M, Zange S, Müller MA (2020). Virological assessment of hospitalized patients with COVID-2019. Nature..

[18] Lo IL, Lio CF, Cheong HH, Lei CI, Cheong TH, Zhong X (2020). Evaluation of SARS-CoV-2 RNA shedding in clinical specimens and clinical characteristics of 10 patients with COVID-19 in Macau. Int J Biol Sci.

[19] Song C, Wang Y, Li W, Hu B, Chen G, Xia P, et al. Detection of 2019 novel coronavirus in semen and testicular biopsy specimen of COVID-19 patients. medRxiv. 2020; 2020.03.31.20042333.

[20] Lu R, Wang J, Li M, Wang Y, Dong J, Cai W. SARS-CoV-2 detection using digital PCR for COVID-19 diagnosis, treatment monitoring and criteria for discharge. medRxiv. 2020; 2020.03.24.20042689.

[21] Xia J, Tong J, Liu M, Shen Y, Guo D (2020). Evaluation of coronavirus in tears and conjunctival secretions of patients with SARS-CoV-2 infection. J Med Virol.

[22] Yuan Y, Wang N, Ou X. Caution should be exercised for the detection of SARS-CoV-2, especially in the elderly. J Med Virol. 2020; n/a(n/a). 10.1002/jmv.25796 32227494

[23] Xiao AT, Tong YX, Zhang S. Profile of RT-PCR for SARS-CoV-2: a preliminary study from 56 COVID-19 patients. Clin Infect Dis. 2020. 10.1093/cid/ciaa460 PMC7188124 32306036

[24] Xu K, Chen Y, Yuan J, Yi P, Ding C, Wu W, et al. Factors associated with prolonged viral RNA shedding in patients with coronavirus disease 2019 (COVID-19). Clin Infect Dis. 2020. 10.1093/cid/ciaa351 PMC7184421 32271376

[25] Zhang W, Du R-H, Li B, Zheng X-S, Yang X-L, Hu B (2020). Molecular and serological investigation of 2019-nCoV infected patients: implication of multiple shedding routes. Emerg Microbes Infect.

[26] Young BE, Ong SWX, Kalimuddin S, Low JG, Tan SY, Loh J (2020). Epidemiologic features and clinical course of patients infected with SARS-CoV-2 in Singapore. JAMA..

[27] Lescure F-X, Bouadma L, Nguyen D, Parisey M, Wicky P-H, Behillil S (2020). Clinical and virological data of the first cases of COVID-19 in Europe: a case series. Lancet Infect Dis.

[28] Jiehao C, Xu J, Lin D, Yang Z, Xu L, Qu Z (2020). A case series of children with 2019 novel coronavirus infection: clinical and epidemiological features. Clin Infect Dis..

[29] He X, Lau EHY, Wu P, Deng X, Wang J, Hao X, et al. Temporal dynamics in viral shedding and transmissibility of COVID-19. medRxiv. 2020; 2020.03.15.20036707.

[30] Hu Z, Song C, Xu C, Jin G, Chen Y, Xu X (2020). Clinical characteristics of 24 asymptomatic infections with COVID-19 screened among close contacts in Nanjing, China. Sci China Life Sci.

[31] Kujawski SA, Wong KK, Collins JP, Epstein L, Killerby ME, Midgley CM, et al. First 12 patients with coronavirus disease 2019 (COVID-19) in the United States. medRxiv. 2020; 2020.03.09.20032896. 10.1038/s41591-020-0877-5 PMC12755114 32327757

[32] Liu F, Xu A, Zhang Y, Xuan W, Yan T, Pan K (2020). Patients of COVID-19 may benefit from sustained lopinavir-combined regimen and the increase of eosinophil may predict the outcome of COVID-19 progression. Int J Infect Dis.

[33] Xu T, Chen C, Zhu Z, Cui M, Chen C, Dai H (2020). Clinical features and dynamics of viral load in imported and non-imported patients with COVID-19. Int J Infect Dis.

[34] Li C, Ji F, Wang L, Wang L, Hao J, Dai M (2020). Asymptomatic and human-to-human transmission of SARS-CoV-2 in a 2-family cluster, Xuzhou. China. Emerg Infect Dis..

[35] Zhang B, Liu S, Dong Y, Zhang L, Zhong Q, Zou Y (2020). Positive rectal swabs in young patients recovered from coronavirus disease 2019 (COVID-19). J Infect..

[36] Xu Y, Li X, Zhu B, Liang H, Fang C, Gong Y (2020). Characteristics of pediatric SARS-CoV-2 infection and potential evidence for persistent fecal viral shedding. Nat Med.

[37] Wu Y, Guo C, Tang L, Hong Z, Zhou J, Dong X (2020). Prolonged presence of SARS-CoV-2 viral RNA in faecal samples. Lancet Gastroenterol Hepatol.

[38] Zheng S, Fan J, Yu F, Feng B, Lou B, Zou Q (2020). Viral load dynamics and disease severity in patients infected with SARS-CoV-2 in Zhejiang province, China, January-March 2020: retrospective cohort study. BMJ.

[39] Lavezzo E, Franchin E, Ciavarella C, Cuomo-Dannenburg G, Barzon L, Del Vecchio C, et al. Suppression of COVID-19 outbreak in the municipality of Vo, Italy. medRxiv. 2020; 2020.04.17.20053157.

[40] Wyllie AL, Fournier J, Casanovas-Massana A, Campbell M, Tokuyama M, Vijayakumar P, et al. Saliva is more sensitive for SARS-CoV-2 detection in COVID-19 patients than nasopharyngeal swabs. medRxiv. 2020; 2020.04.16.20067835.

[41] Amirian ES (2020). Potential fecal transmission of SARS-CoV-2: current evidence and implications for public health. Int J Infect Dis.

[42] Kojima N, Turner F, Slepnev V, Bacelar A, Deming L, Kodeboyina S, et al. Self-collected oral fluid and nasal swabs demonstrate comparable sensitivity to clinician collected nasopharyngeal swabs for covid-19 detection. medRxiv. 2020; 2020.04.11.20062372. 10.1093/cid/ciaa1589 PMC7665422 33075138

[43] Gates B. 2020. https://www.youtube.com/watch?v=Xe8fIjxicoo. Accessed 16 June 2020.

