# Supplementary material for: At what times during infection is SARS-CoV-2 detectable and no longer detectable using RT-PCR-based tests? A systematic review of individual participant data
Source: BMC Med. 2020 Nov 4;18:346. doi: 10.1186/s12916-020-01810-8 (PMC7609379; doi:10.1186/s12916-020-01810-8)
Supplement: Supplementary file 1 — Additional file 1. Including additional tables and figures: search details, QUADAS-2 adaption, anatomical sample size details, risk of bias by article, percentage positive and negative RT-PCR results by sample for days since symptom onset and days since hospitalisation, time to undetectable virus in faecal and respiratory tract. [file 12916_2020_1810_MOESM1_ESM.pdf]

Additional File 1

Contents

Table S1: Search strings and methods........2
Table S2: QUADAS-2 adaptation for diagnostic study using longitudinal sampling........3
Table S3: Data extraction additional methods........5
Table S4: Summary of sample sites and tests........6
Table S5: Risk of bias by article........7
Figure S6: Percentage of positive and negative RT-PCR test results since symptom onset........8
Figure S7: Percentage of positive and negative RT-PCR test results since hospital admission........9
Figure S8: Time to undetectable virus in respiratory tract and faecal sampling........10

TABLE S1: SEARCH STRINGS AND METHODS

Search strings	
Database	Search terms
Medline (via PubMed)	((coronavirus*[Title] OR coronovirus*[Title] OR coronoravirus*[Title] OR coronaravirus*[Title] OR corono-virus*[Title] OR corona-virus*[Title] OR “Coronavirus”[Mesh] OR “Coronavirus Infections”[Mesh] OR “Wuhan coronavirus” [Supplementary Concept] OR "Severe Acute Respiratory Syndrome Coronavirus 2"[Supplementary Concept] OR COVID-19[All Fields] OR CORVID-19[All Fields] OR “2019nCoV”[All Fields] OR “2019-nCoV”[All Fields] OR WN-CoV[All Fields] OR nCoV[All Fields] OR “SARS-CoV-2”[All Fields] OR HCoV-19[All Fields] OR “novel coronavirus”[All Fields])) AND (((reverse transcript*[Text Word] AND ("polymerase chain reaction"[Text Word] OR pcr)[Text Word]) OR (rt-pcr[Text Word] OR "rt pcr"[Text Word])) OR ("Reverse Transcriptase Polymerase Chain Reaction"[Mesh]))
LitCOVID	(reverse transcript* AND ("polymerase chain reaction" OR pcr)) OR rt-pcr OR "rt pcr"
medRxiv	(coronavirus OR covid-19) AND (pcr OR "polymerase chain reaction") AND (clearance OR detect OR detection OR detected)<br>"(coronavirus OR covid-19) AND (pcr OR "polymerase chain reaction") AND (diagnosis OR diagnostic)"
Additional details of search methods	
Search strings were designed and conducted subsequently in Medline via PubMed, LitCOVID and medRxiv by an experienced information specialist (NR). We additionally included references identified by COVID-19:NIHR living map of living evidence (http://eppi.ioe.ac.uk/COVID19_MAP/covid_map_v4.html), COVID-19 Living Evidence (https://ispmbern.github.io/covid-19/living-review/) with a volunteer citizen science team, "The Virus Bashers".<br>We additionally included references identified by COVID-19:NIHR living map of living evidence (http://eppi.ioe.ac.uk/COVID19_MAP/covid_map_v4.html), COVID-19 Living Evidence (https://ispmbern.github.io/covid-19/living-review/), and searched citation lists from identified articles (including those from Google Scholar, and from systematic reviews) and also articles and references forwarded by colleagues. The search was to 24th April 2020 inclusive for the databases and the COVID-19 Living Evidence database. Preliminary screening of titles from PubMed, LitCOVID and medRxiv searches (NR) and preliminary screening of full text articles by a volunteer citizen science team, "The Virus Bashers", (COVID-19 Living Evidence database) were followed full article screening by a single author (SM) with reference to second reviewers as needed (BS, ZZ, CH, JP). The volunteer citizen science team screened articles for those including IPD for RT-PCR testing where participants were tested on more than one occasion. They were provided with training information with examples of tables and figures of how IPD data can be reported, weblinks to articles, and how to access supplementary material for each article. An experienced professional volunteer organiser co-ordinated, supported and fielded preliminary queries (SH) and referred further queries for clarification (SM).	

TABLE S2: QUADAS-2 INCLUDING EXTENSION FOR DIAGNOSTIC STUDY USING LONGITUDINAL SAMPLING

There is no available tool to assess the accuracy of diagnostic studies that use longitudinal sampling. We therefore developed our own tool using the QUADAS-2[6] tool as a starting point. QUADAS-2 is designed for diagnostic test accuracy studies, whereas we required a tool to understand the potential for bias in sampling methods including timing of sampling. We considered the domains and signalling questions included in QUADAS-2 together with areas where bias may arise in diagnostic studies using longitudinal sampling. For these types of study, where participants are selected because they have SARS-CoV-2 infection, issues of reference standard (how SARS-CoV-2 infection was diagnosed) are inextricably linked with how participants are selected for inclusion. We therefore considered these issues in combination as part of domain 1 (Participant Selection). Signalling questions and evaluation of risk of bias were devised (SM) and refined after consultation (BS, PW, RW) during the protocol stage of this review. Signalling questions were pre-specified prior to data extraction, but the order and refinement of the wordings of some questions were adjusted during the review (PW, RW). Assessment of applicability was not assessed as our review had a very broad review question and so assessing whether the studies matched the review question was not appropriate.

**Domain 1: Participant selection**		
Description	Describe patient population, and any methods used to select patients to receive more than one PCR test<br>Describe how COVID-19 diagnosis was confirmed.	
Risk of bias	SQ1: Were all participants in the cohort tested with longitudinal PCR tests?	– Yes if participants were enrolled in a way so they would remain typical of the main clinical population<br>– No if participants were selected as a specialised group e.g. where PCR test disagrees/agrees with other tests such as chest x-ray<br>– Unclear if insufficient data are reported to permit a judgment
	SQ2: Did the study avoid inappropriate exclusions?	– Yes if participants were enrolled in a way so they would remain typical of the main clinical population<br>– No if participants were selected as a specialised group e.g. where PCR test disagrees/agrees with other tests such as chest x-ray<br>– Unclear if insufficient data are reported to permit a judgment
	SQ3: Did the study allow diagnosis of COVID-19 without requiring confirmation by a positive PCR test result?	– Yes if diagnosis of COVID did not require a positive PCR test result<br>– No if a positive PCR test result was required<br>– Unclear if this information is unclear
Summary judgement of risk of bias	Could the selection of participants have introduced bias?	– Low: If SQ1 & SQ2 & SQ3 are `Yes'<br>– High: If any of SQ1 & SQ2 & SQ3 are `No'<br>– Unclear: If insufficient data are reported to permit a judgment
**Domain 2: Index test**		
Description	Describe PCR test methods	
Risk of bias	SQ4: Was the timing of PCR testing pre-specified? (frequency of testing)	– Yes if participants were tested according to a fixed protocol e.g. every 2 days and then 24 hrs after a negative test result.<br>– No if participants test results were reported without a regular time interval or protocol. Testing in these situations is often prompted by results of previous tests.<br>– Unclear if insufficient data are reported to permit a judgment

	SQ5: Were the samples used for PCR testing pre-specified?	– Yes if samples for testing were the same for all participants or were pre-specified e.g. all participants were asked to provide nasopharyngeal, saliva and stool samples for testing<br>– No if samples for testing were chosen differently or without protocol (e.g. different participants had different samples taken or from different samples at different times)<br>– Unclear if insufficient data are reported to permit a judgment
Summary judgement of risk of bias	Could the conduct of the index test have introduced bias?	– Low if SQ6 & SQ7 are both `Yes'<br>– High if either or both of SQ6 or SQ7 are `No'<br>– Unclear if insufficient data are reported to permit a judgment
**Domain 3: Flow and timing**		
Description	Describe any details on how patients were followed up for PCR testing	
Risk of bias	SQ6: Did all participants continue to receive PCR tests irrespective of whether they remained in hospital?	– Yes if testing stopped when a participant was discharged from hospital<br>– No if the partcipants continued testing was not influenced by easy access to patients (e.g. whether they remained in hospital)<br>– Unclear if insufficient data are reported to permit a judgment
	SQ7: Did participants with a single negative PCR test result receive the same further tests as those with a positive test result?	– Yes if participant samples for testing were the same for patients or pre-specified e.g. all participants were asked to give samples for testing from nasopharyngeal, saliva and stool<br>– No if participants samples for testing were apparently chosen differently or without protocol (e.g. different participants had different samples taken or different samples at different times)<br>– Unclear if insufficient data are reported to permit a judgment
	SQ8.Were results available for all participants who had longitudinal PCR results?	– Yes if all participant data could be extracted or was available for the study<br>– No if some participants did not have full PCR test results reported<br>– Unclear if insufficient data are reported to permit a judgment
Summary judgement of risk of bias	Could the participant flow have introduced bias?	– Low: If SQ8 & SQ9 & SQ12 are `Yes'<br>– High: If any of SQ8 & SQ9 & SQ12 are `No'<br>– Unclear: If insufficient data are reported to permit a judgment

TABLE S3: DATA EXTRACTION ADDITIONAL METHODS

**RT-PCR test result conversion to binary results**<br>IPD RT-PCR results were extracted from each article and afterwards converted to binary results ("positive" or "negative") to facilitate combination across all studies. For articles reporting viral load, values above a stated threshold value were defined as positive. In our review, thresholds were extracted for each article and used to define the positive test results for that study; usually threshold values were at 2 or 3 RNA copies/ml. Where a threshold was not reported, a value of 2 was used. For articles reporting viral load using cycle threshold values (Ct), Ct values below the stated threshold indicate a positive test. Thresholds reported by individual articles were used, usually corresponding to 38 or 40 cycles. Where no threshold was reported, the results in the article were examined and a threshold of 38 or 40 used as appropriate checking consistency with text descriptions of test results. For articles reporting a difference in cycle threshold (Difference in Ct), a positive test result was defined using a threshold of zero.
**Data analysis**<br>Days since symptom onset and days since hospital admission were calculated from reported IPD. Data were presented collated across 5-day time intervals for each sample method, with longer times grouped within the longest time interval. Intermediate test results reported in the individual articles were classified as positive for data analysis. As analysis aimed to investigate the percentage of test results within the time intervals that were positive, more than one result per participant could be included within the same time interval if sufficient data were available. 95% CI were calculated for proportions.<br>For comparison of duration of positive RT-PCR from respiratory tract (RT) and faecal samples, analysis and graphical presentation was restricted to participants sampled by both methods. A scatterplot was produced to display duration in days of virus detection for all participants with reported IPD results tested with both faecal and RT sampling. Kaplan-Meier (KM) and log rank tests by site of sampling for duration of virus detection were based on participants positive with both faecal and RT tests.<br>Intermittent false negative results were defined as negative RT-PCR results which are followed by positive RT-PCR results at a later time in the same participant. For duration of detectable virus, the latest positive RT-PCR result in that sample site was used for each participant. When two anatomical sampling sites were compared, some participants were tested until negative RT-PCR test results were obtained for both samples, labelled as "tested until negative" on figures, whereas participants where one or both samples had positive results at the last time of testing, these are labelled as "still positive" Data analysis was conducted using STATA (14.2 StataCorp LP, Texas, USA).
**Sampling site**<br>Nasopharyngeal, saliva, sputum were used where clearly reported. Other URT includes samples reported in articles as: nasal, mixed nasal and throat, oral, pharyngeal or upper respiratory tract. For pharyngeal sampling it was not clear if this was nasopharyngeal or oropharyngeal. Other LRT includes sampling reported as lower respiratory tract or one article including pleural sampling. We grouped together serum, plasma and blood; oropharyngeal and throat; stool and anal swabs. BAL, tracheal aspirate and pleural fluid were reported by few studies and grouped as LRT for analysis. Saliva and sputum are presented separately.

TABLE S4: SUMMARY OF SAMPLE SITES AND TESTS

Sample site	Number of PCR tests	N (%) articles
Faeces	266	13 (41)
Other URT	478	11 (34)
Nasopharyngeal	242	10 (31)
Throat	193	9 (28)
Sputum	158	6 (19)
Blood	143	6 (19)
Urine	33	4 (13)
Saliva	23	2 (6)
Conjunctiva	34	2 (6)
Other LRT	14	2 (6)
Unspecified	35	1 (3)

Footnote
Semen and testicular samples in one article were not extracted

TABLE S5: RISK OF BIAS BY ARTICLE

Study ID	Participants	Index test (RT-PCR)	Flow and timing
Cai 2020	H	U	U
Chang 2020	H	L	H
Chen 2020a	H	H	H
Chen 2020b	H	H	H
He 2020	H	L	L
Hu 2020a	H	L	L
Hu 2020b	H	L	L
Jiehao 2020	H	U	U
Kujawski 2020	H	L	L
Lavezzo 2020	H	L	H
Lescure 2020	H	L	L
Li 2020	H	L	L
Liu 2020a	H	U	L
Liu 2020b	H	U	H
Lo 2020	H	L	L
Lu 2020	H	U	H
Song 2020	H	U	H
To 2020	H	U	H
Wolfel 2020	L	H	H
Wu 2020	H	L	L
Wyllie 2020	H	L	H
Xia 2020	H	L	L
Xiao 2020	H	L	L
Xu 2020a	H	L	L
Xu 2020b	H	L	L
Yang 2020	H	L	H
Young 2020	H	L	L
Yuan 2020	H	L	L
Zhang 2020a	H	L	L
Zhang 2020b	H	L	L
Zheng 2020c	H	L	L
Zhou 2020	H	U	U

Footnote: H=high risk of bias, L=low risk of bias, U=unclear risk of bias

FIGURE S6: PERCENTAGE OF POSITIVE AND NEGATIVE RT-PCR TEST RESULTS SINCE SYMPTOM ONSET

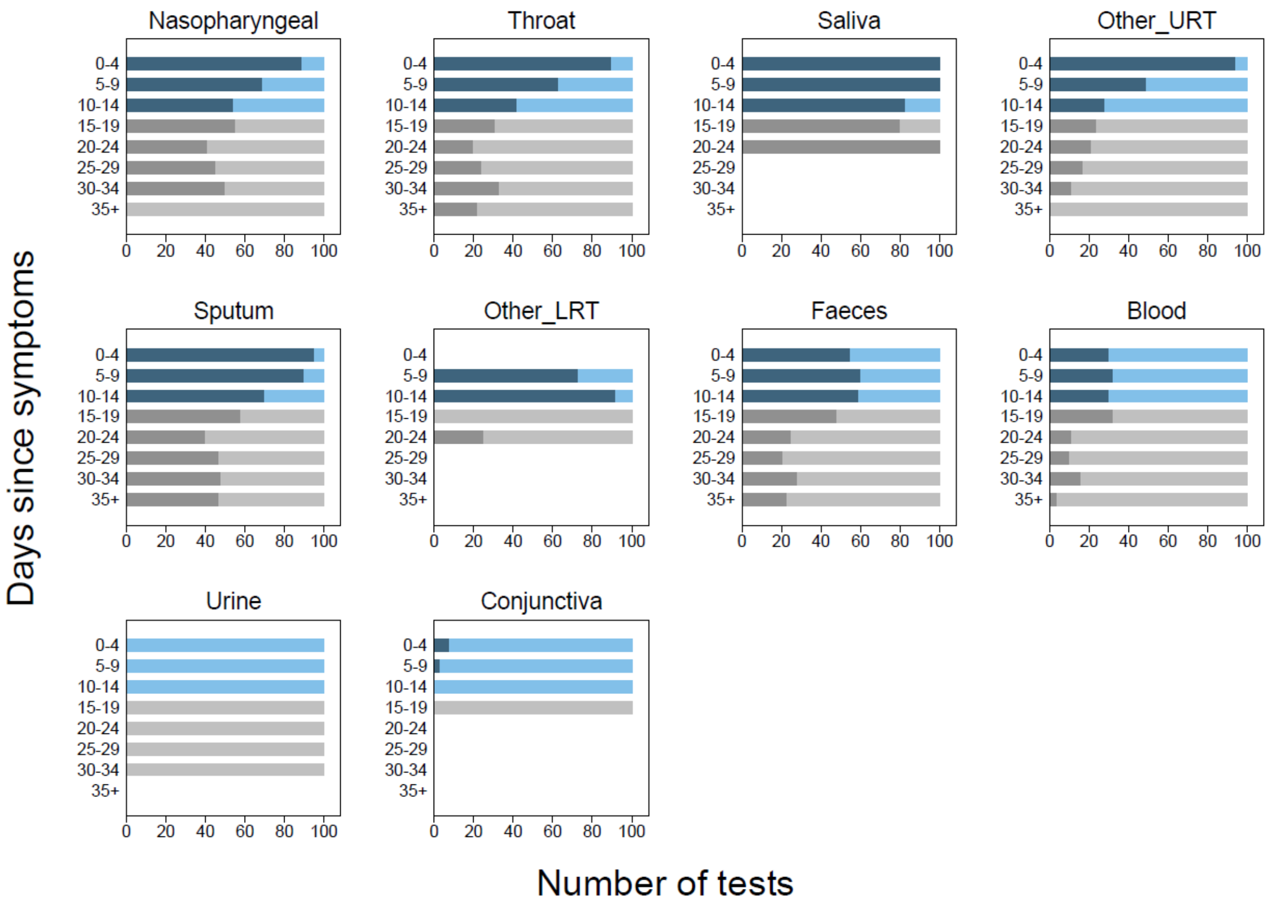

Figure S6: Percentage of positive and negative RT-PCR test results since symptom onset

Each panel shows a separate site used in participant sampling. Other_URT includes samples reported in articles as: nasal, mixed nasal and throat, oral, pharyngeal, or upper respiratory tract. For pharyngeal sampling it was not clear if this was nasopharyngeal or oropharyngeal. Other_LRT includes sampling reported as lower respiratory tract or one article including pleural fluid sampling.

Each panel shows 5-day time periods since the onset of symptoms: 0-4 days, 5-9 days, 10-14 days, 15-19 days, 20-25 days, 26-30 days, 31-34 days, 35 to max days.

The numbers of positive RT-PCR tests are shown as dark blue bars and dark grey bars between 0 to 14 days and 15 to 40 days respectively, and the number of negative RT-PCR results are shown similarly as light blue bars and light grey bars. Different colours are used before and after 15 days to indicate caution, as after 15 days testing is enriched in more severely ill participants. This figure should be read in conjunction with figure 2 which shows how the number of data points underlying the percentages varies.

FIGURE S7: PERCENTAGE OF POSITIVE AND NEGATIVE RT-PCR TEST RESULTS SINCE HOSPITAL ADMISSION

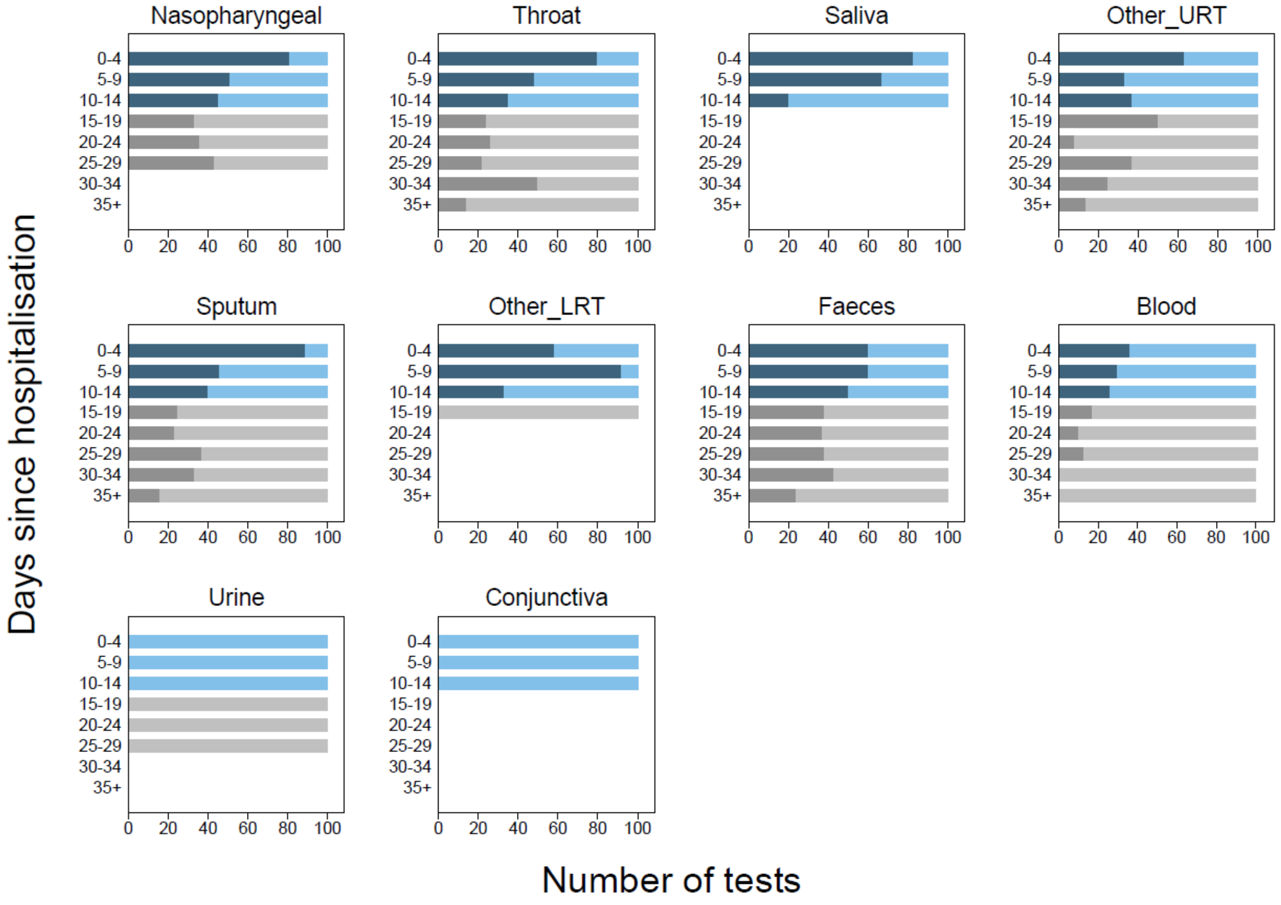

Figure S7: Percentage of positive and negative RT-PCR test results since hospital admission

Each panel shows a separate site used in participant sampling. Other_URT includes samples reported in articles as: nasal, mixed nasal and throat, oral, pharyngeal, or upper respiratory tract. For pharyngeal sampling it was not clear if this was nasopharyngeal or oropharyngeal. Other_LRT includes sampling reported as lower respiratory tract or one article including pleural fluid sampling.

Each panel shows 5-day time periods since the hospital admission: 0-4 days, 5-9 days, 10-14 days, 15-19 days, 20-25 days, 26-30 days, 31-34 days, 35 to max days.

The numbers of positive RT-PCR tests are shown as dark blue bars and dark grey bars between 0 to 14 days and 15 to 40 days respectively, and the number of negative RT-PCR results are shown similarly as light blue bars and light grey bars. Different colours are used before and after 15 days to indicate caution, as after 15 days testing is enriched in more severely ill participants. This figure should be read in conjunction with figure 2 which shows how the number of data points underlying the percentages varies.

FIGURE S8: TIME TO UNDETECTABLE VIRUS IN RESPIRATORY TRACT AND FAECAL SAMPLING

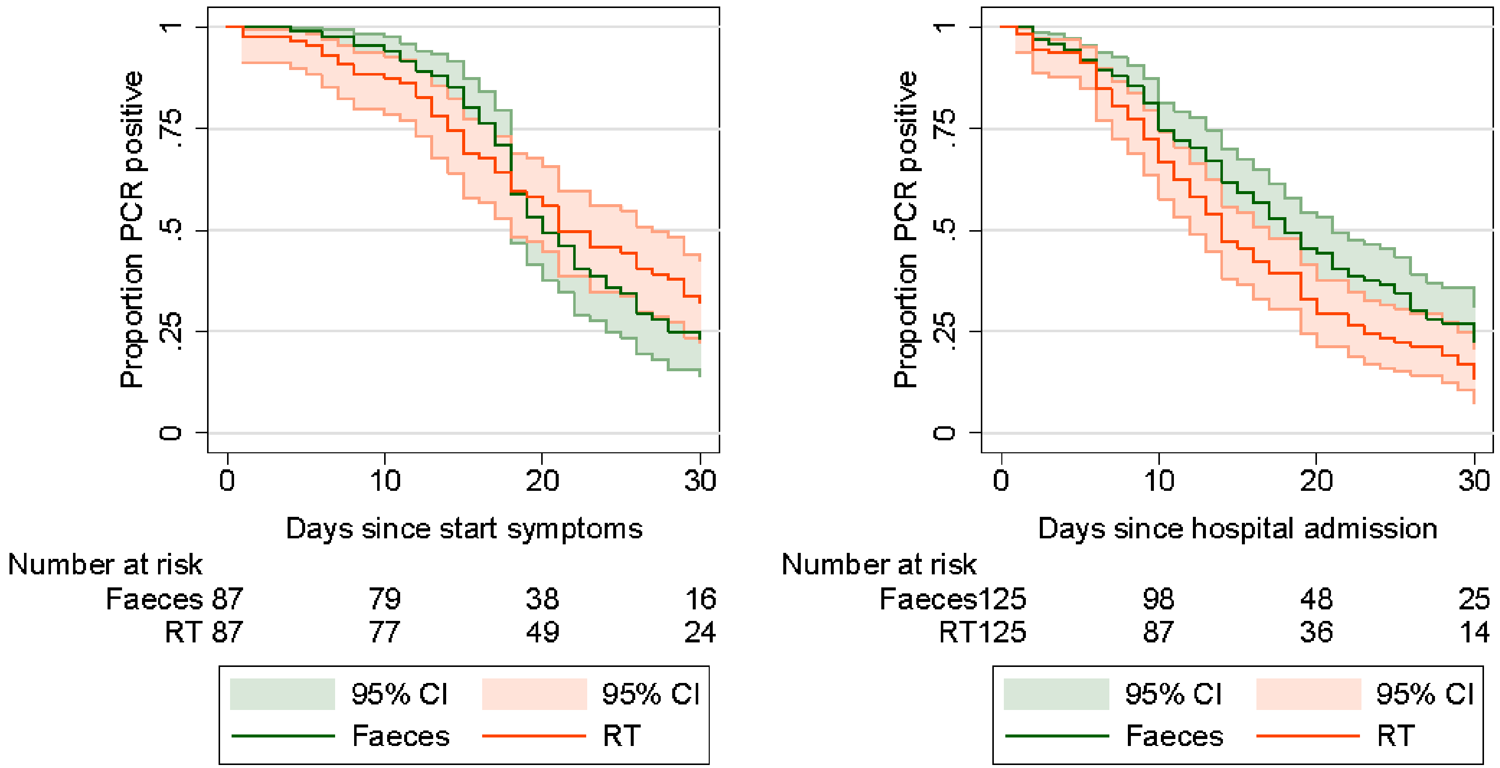

Figure 8: Time to undetectable virus in respiratory tract and faecal sampling

Kaplan-Meier with 95% confidence intervals and number at risk. Restricted to participants with both sampling methods. (A) Days since symptom onset (B) Days since hospitalisation. RT=respiratory tract sample (upper or lower respiratory tract)
